# F100. FACTOR STRUCTURE OF THE CANNABIS EXPERIENCES QUESTIONNAIRE IN A FIRST-EPISODE PSYCHOSIS SAMPLE

**DOI:** 10.1093/schbul/sby017.631

**Published:** 2018-04-01

**Authors:** Michael Birnbaum, Luca Pauselli, Sean Cleary, Claire Ramsey Wan, Michael Compton

**Affiliations:** 1 North Shore-LIJ; 2 College of Physicians and Surgeons, Columbia University, New York State Psychiatric Institute; 3 The George Washington University; 4 Cambridge Health Alliance; 5 College of Physicians and Surgeons, Columbia University

## Abstract

**Background:**

The Cannabis Experiences Questionnaire (CEQ) was developed to measure the subjective experiences of cannabis use both during and after intoxication. Despite the need to better understand the nature of the complex and significant relationship between cannabis use and early psychosis, this questionnaire has rarely been used in individuals with first-episode psychosis.

**Methods:**

We conducted a set of factor analyses using CEQ data from 194 first-episode psychosis patients who used cannabis, in order to uncover the underlying factor structure of the questionnaire and thus the overarching types of psychological experiences during/after using cannabis in young people with psychotic disorders.

**Results:**

Confirmatory factor analyses were performed on the 2 full-scale CEQ factor structures identified in the literature and neither model fit the data within acceptable levels. Using all 56 CEQ items, an exploratory factor analysis (EFA) model was fit with an oblique rotation. Models with 3, 4, and 5 factors were further explored to identify underlying factors. The final 4-factor EFA model provided the best fit. It included 47 items (3 items had multiple loadings and 6 items did not load on any factor), with names given, based on item composition, as follows: Factor 1 (Distortions of Reality and Self-Perception) included 18 items (α = 0.89), Factor 2 (Euphoria Effects) included 16 items (α = 0.89), Factor 3 (Slowing and Amotivational Effects) included 7 items (α = 0.81), and Factor 4 (Anxiety and Paranoia Effects) included 6 items (α = 0.79).

**Discussion:**

Our derived factor structure differed from those stemming from previous EFAs using different samples (eg, healthy individuals with varying degrees of schizotypy). The inconsistency might be best explained by the different populations sampled, ranging from healthy individuals who have smoked cannabis at least once to individuals with schizophrenia who smoked it regularly. Specifically, differences could be related to variations in how cannabis affects healthy individuals as well as those with schizotypy, as opposed to those with emerging or frank psychosis. Elucidating the underlying factor structure of the CEQ in first-episode psychosis samples could help researchers move towards a deeper understanding of the types of experiences associated with cannabis intoxication among young adults with first-episode psychosis and could inform the development of programs designed to reduce use, improve the course of illness, and possibly delay or prevent the onset of psychotic symptoms in those at risk.

